# Supplementary material for: Migratory dendritic cells acquire and present lymphatic endothelial cell-archived antigens during lymph node contraction
Source: Nat Commun. 2017 Dec 11;8:2034. doi: 10.1038/s41467-017-02247-z (PMC5725486; doi:10.1038/s41467-017-02247-z)
Supplement: Supplementary file 1 — Supplementary Information [file 41467_2017_2247_MOESM1_ESM.pdf]

Supplementary Figures.

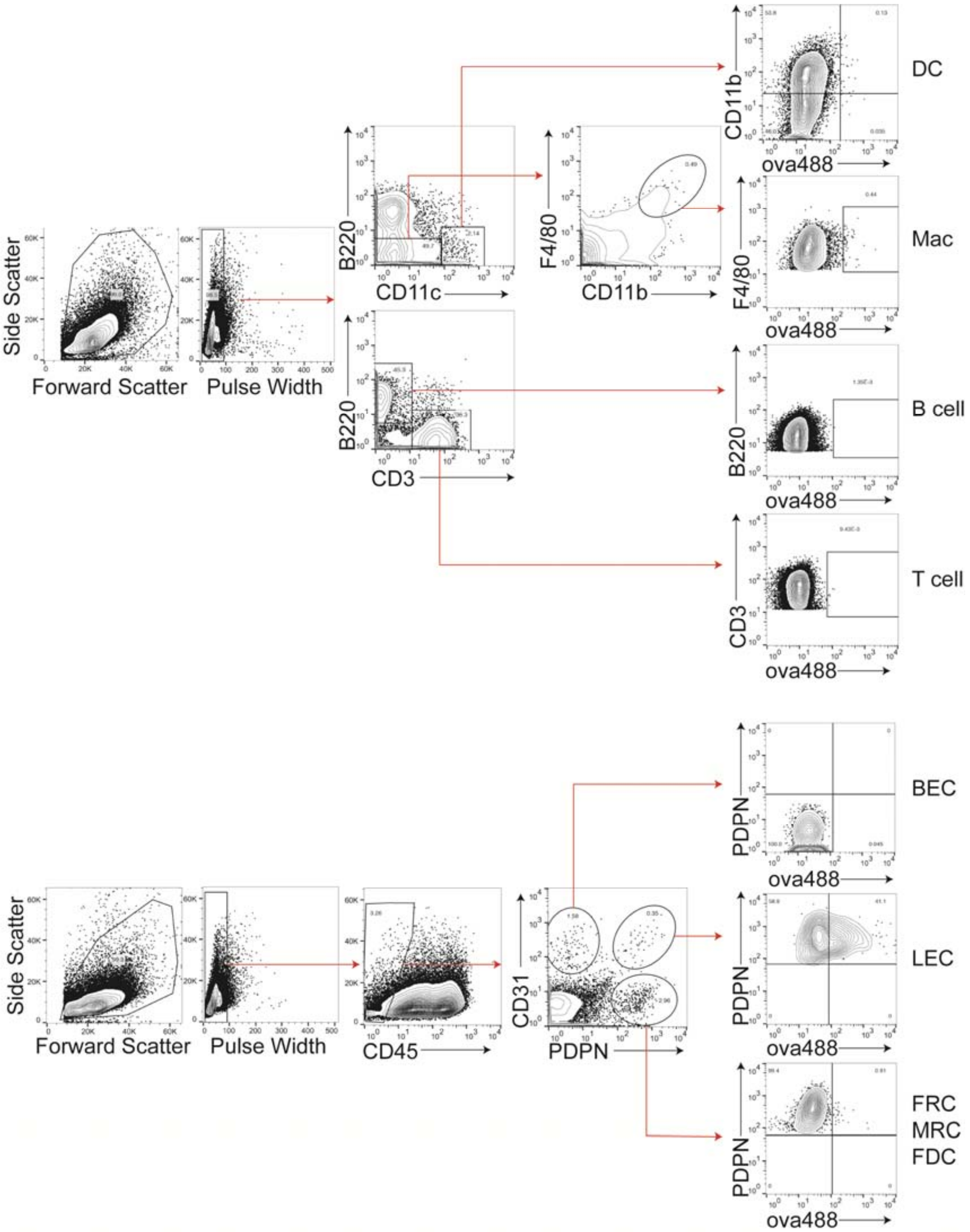

Supplementary figure 1. Gating strategy for hematopoietic and stromal cell subsets outlined in figure 1. Red arrows indicate the boxed cells are gated into the next plot. Shown are dendritic cell (DC), macrophage (Mac), B cell, T cell, blood endothelial cell (BEC), lymphatic endothelial cell (LEC), fibroblastic reticular cell (FRC), marginal reticular cell (MRC), follicular dendritic cell (FDC).

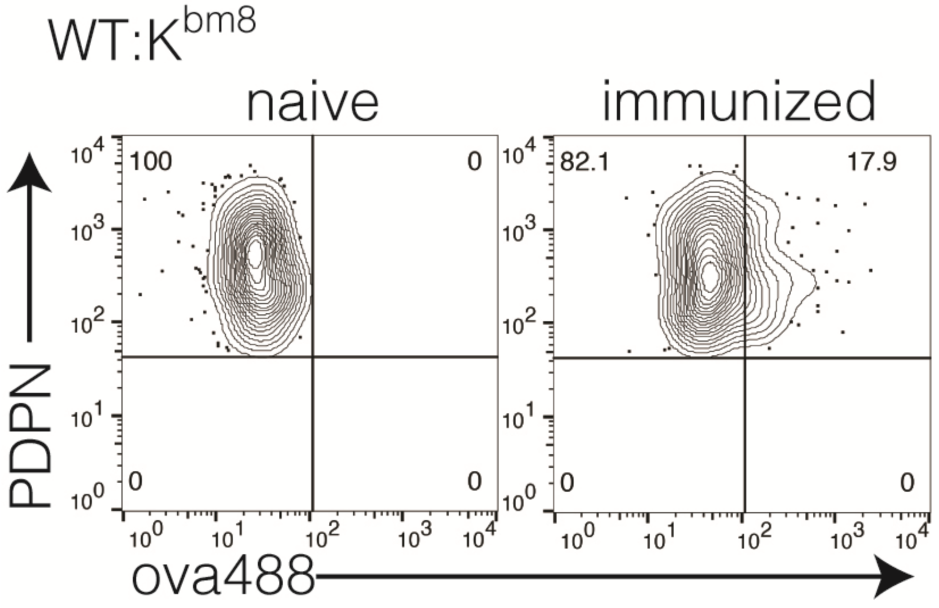

Supplementary figure 2. Antigen archiving occurs normally in bone marrow chimeras. Naïve or 10μg ovalbumin conjugated to alexafluor 488 with 2μg polyI:C and 2μg anti-CD40 into each subcutaneous site (footpad, flank, scruff) was used to immunize WT:K^{bm8} bone marrow chimeras. Mice were euthanized after 2.5 weeks. Shown are antigen$^+$ CD45$^-$, CD31$^+$, PDPN$^+$, cells. Experiment was performed twice with 3 mice per group. Shown is a representative image of antigen archiving.

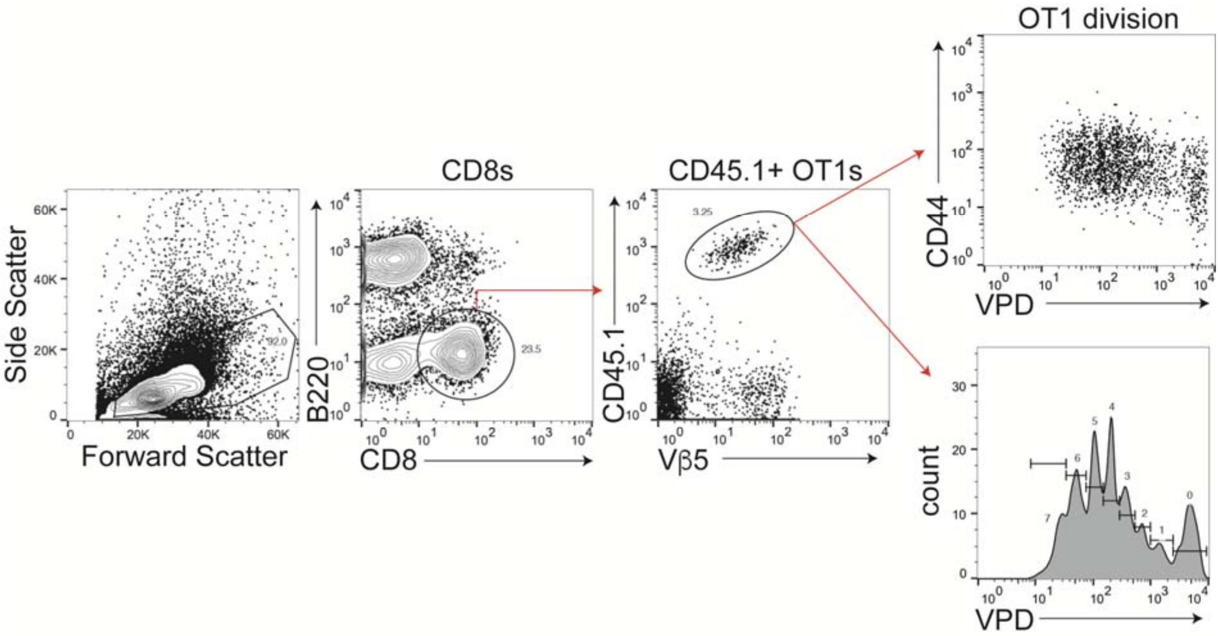

Supplementary figure 3. Gating strategy for OT1 transfer system. Red arrows indicate the boxed cells are gated into the next plot.

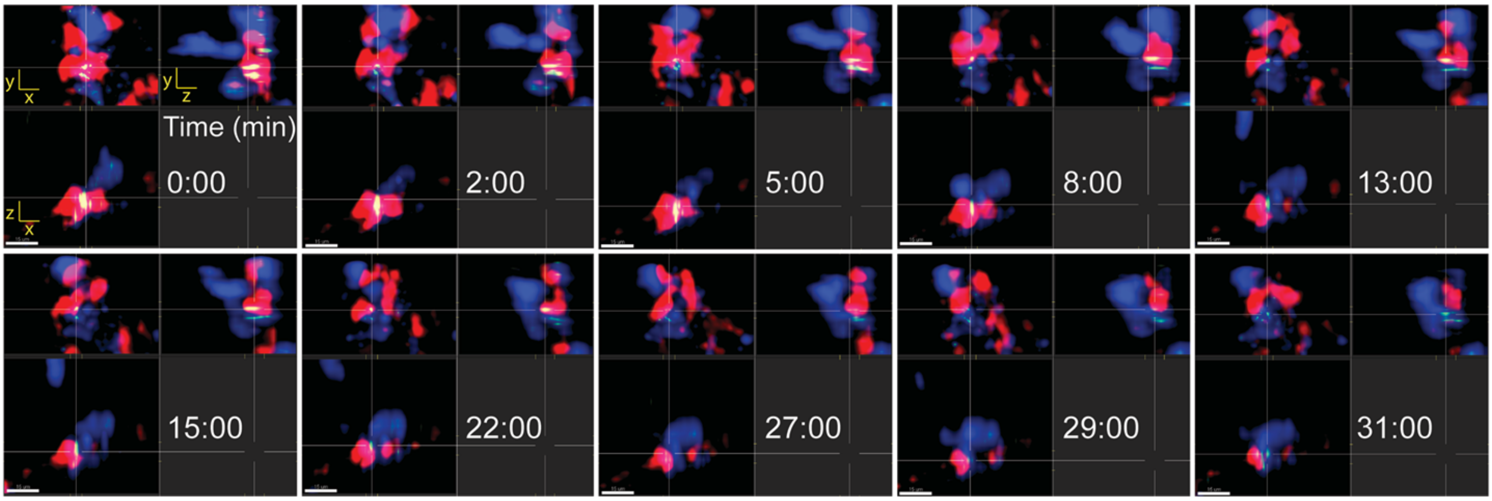

Supplementary figure 4. Lymphatic endothelial cells exchange antigen with dendritic cells. Orthogonal views of images in figure 2 from a multiphoton image of an explanted lymph node demonstrating antigen (green) in the center of the lymphatic endothelial cell (red) at time-point 0:00 and ending with antigen in the center of the dendritic cell (blue) at time-point (31:00). Scale bar is 15 microns. X-y (top left), y-z (top right), and x-z (bottom left) in each panel indicate the direction of the plane of view.

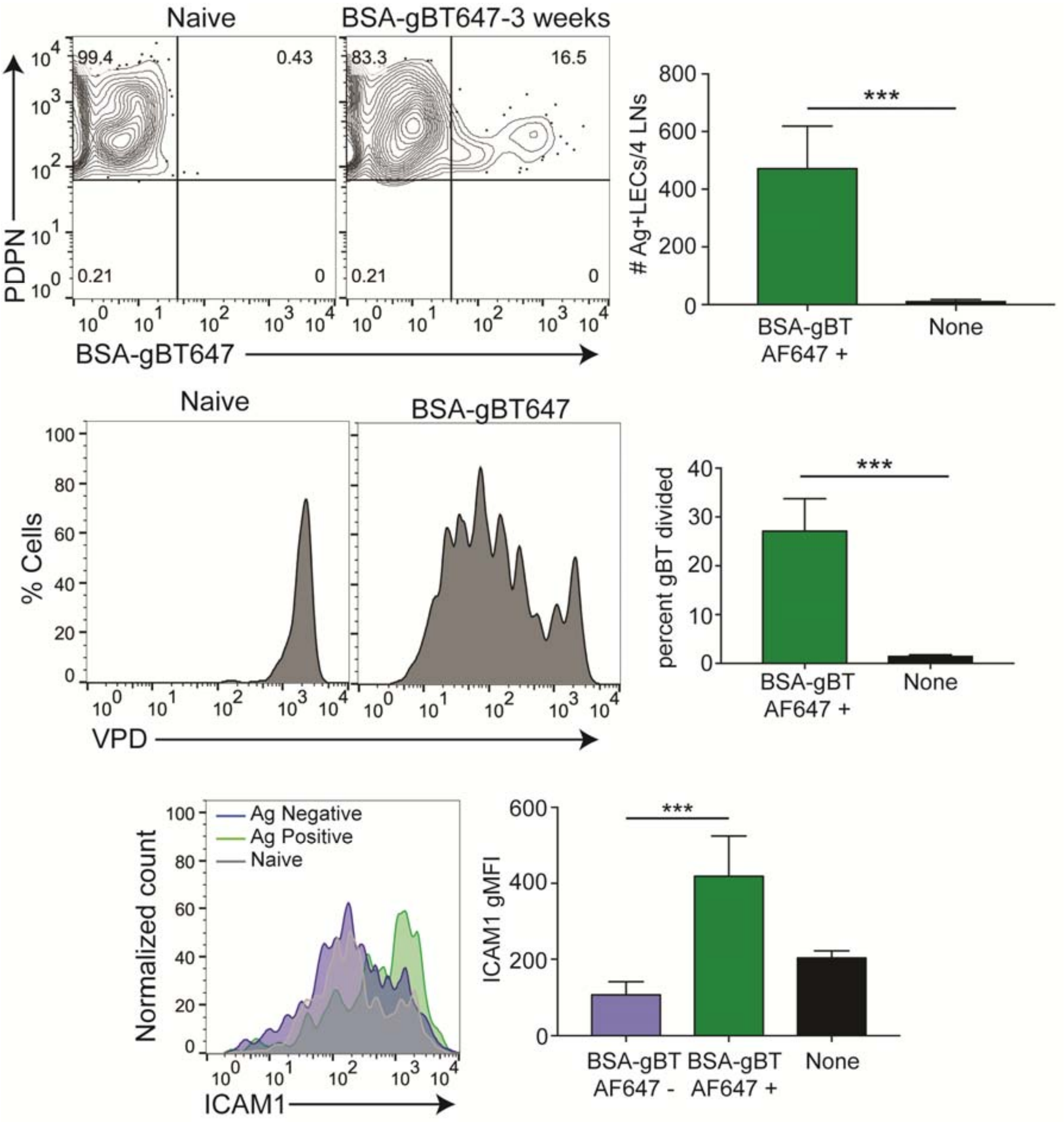

Supplementary figure 5. Antigen archiving, exchange, and presentation occurs with different antigens. Bovine serum albumin (BSA) protein was conjugated to SSIEFARL peptide using maleimide conjugation and then labeled with Alexa Fluor 647 (BSA-gBT647). Mice were immunized with BSA-gBT647+polyI:C and αCD40 as described in figure 2. Two or three weeks later gBT T cell receptor transgenic CD8 T cells, which recognize SSIEFARL, were labeled with VPD and transferred into immunized mice. Shown are gated lymphatic endothelial cells (LEC) and gBTs T cell receptor transgenic T cells as in supplementary figures 1 and 3. ICAM1 expression of antigen positive versus antigen negative LECs or naïve LECs was evaluated by flow cytometry and geometric mean fluorescence intensity (gMFI) was quantified. Error bars represent the standard error of the mean. An unpaired t-test was performed to calculate significance where 3 asterisks represent a p-value <0.0001. Three mice per group were used and experiment was repeated 3 times.

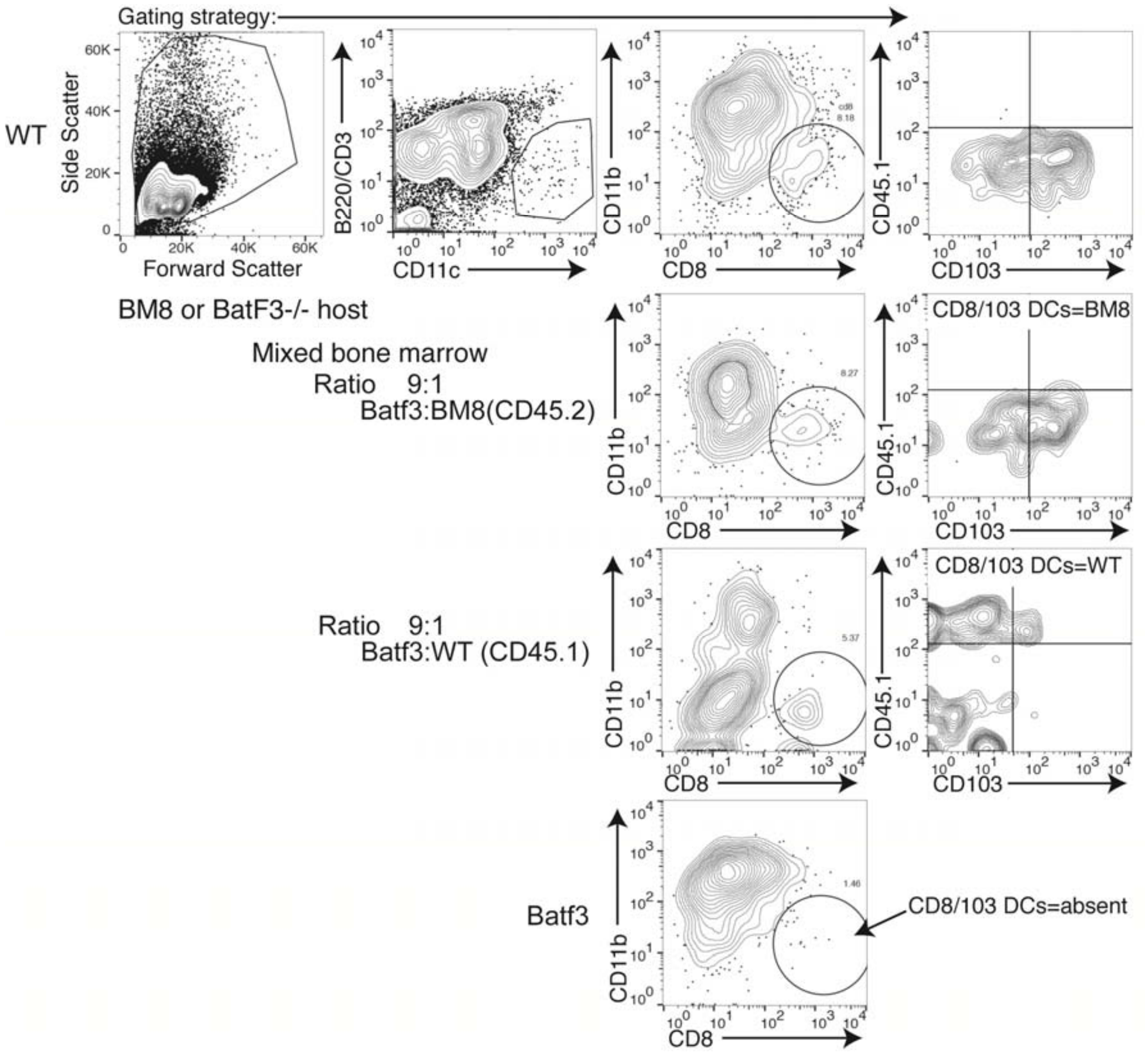

Supplementary figure 6. Gating strategy for dendritic cells described in figure 6. WT bone marrow used was congenically labeled as CD45.1 to evaluate reconstitution of bone marrow into the BatF3 dependent dendritic cell compartment.

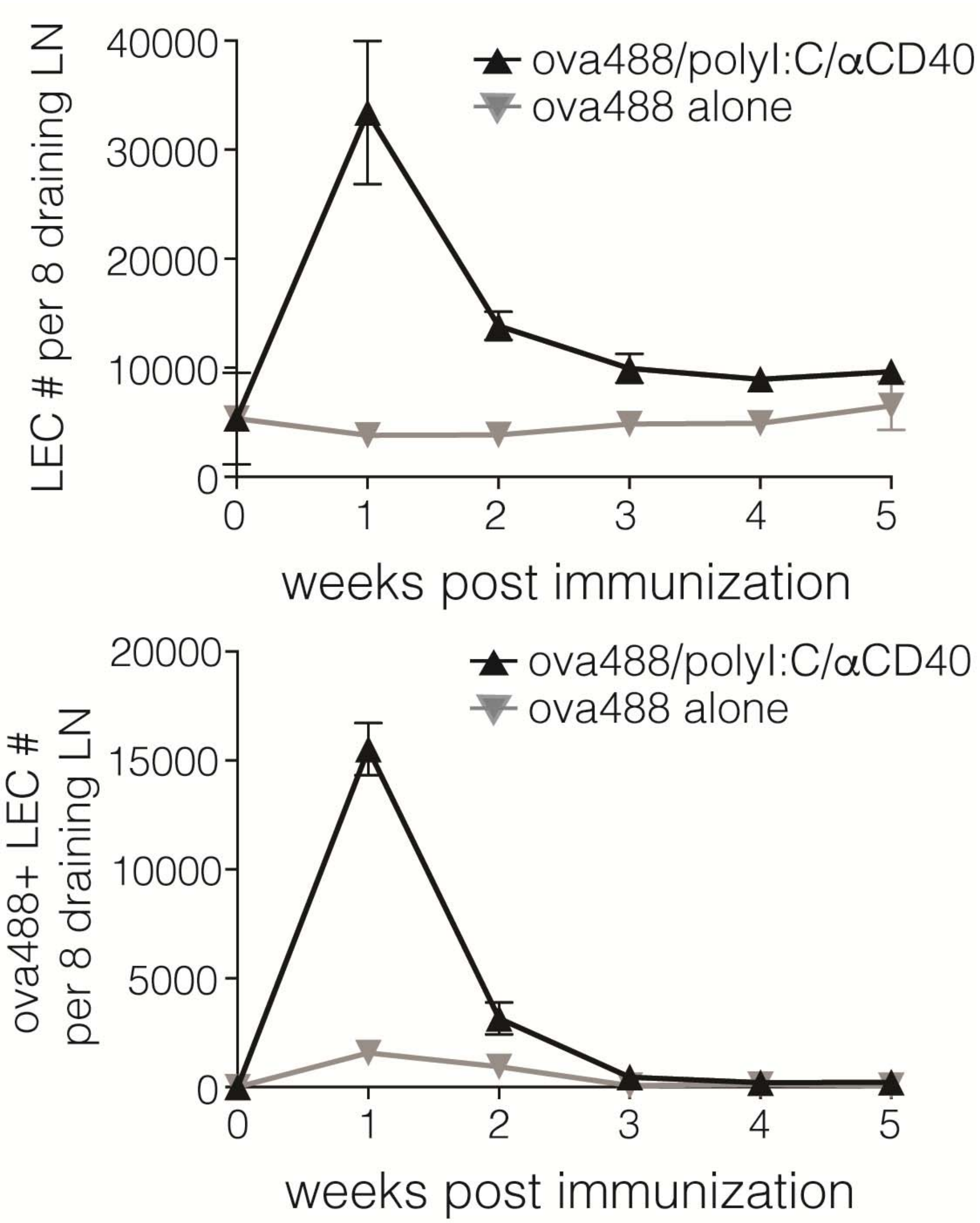

Supplementary figure 7. Expansion and contraction of the lymph node occurs following subunit vaccination. Wild-type mice were immunized weekly for 5 weeks with ovalbumin conjugated to alexafluor 488 (ova488) (10μg) polyI:C/ αCD40 (2μg each) per site (footpads, flanks, scruff) or just ovalbumin-488 alone and the number of lymphatic endothelial cells (LEC) and ova488+ lymphatic endothelial cells from the draining lymph nodes was determined. Three mice per group per time-point were used and experiment was repeated three times. Error bars represent standard error of the mean.

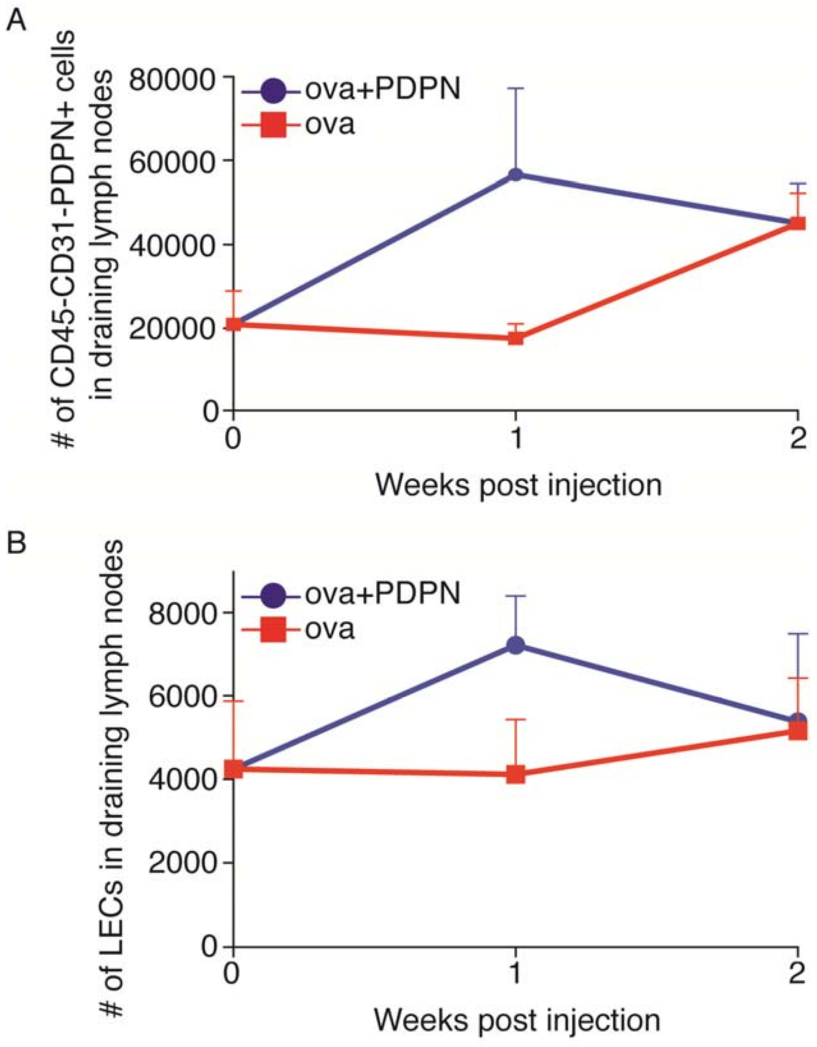

Supplementary figure 8. Anti-podoplanin treatment results in increased numbers of fibroblastic reticular cells and lymphatic endothelial cells. A. The total number of $CD45^-$, $CD31^-$, $PDPN^+$ cells (FRC) following 2-100 μg injections of anti-PDPN at day 0 and day 3 (ova+PDPN) or no PDPN (ova) were quantified at 0, 1, and 2 weeks. B. As in A, except $CD45^-$, $CD31^+$, $PDPN^+$ (LEC). Error bars are mean + standard error and statistical analysis was calculated from groups described in figure 8. Gating was performed as in Supplementary figure 1, bottom panel. Three mice per group were used and experiment was repeated twice.

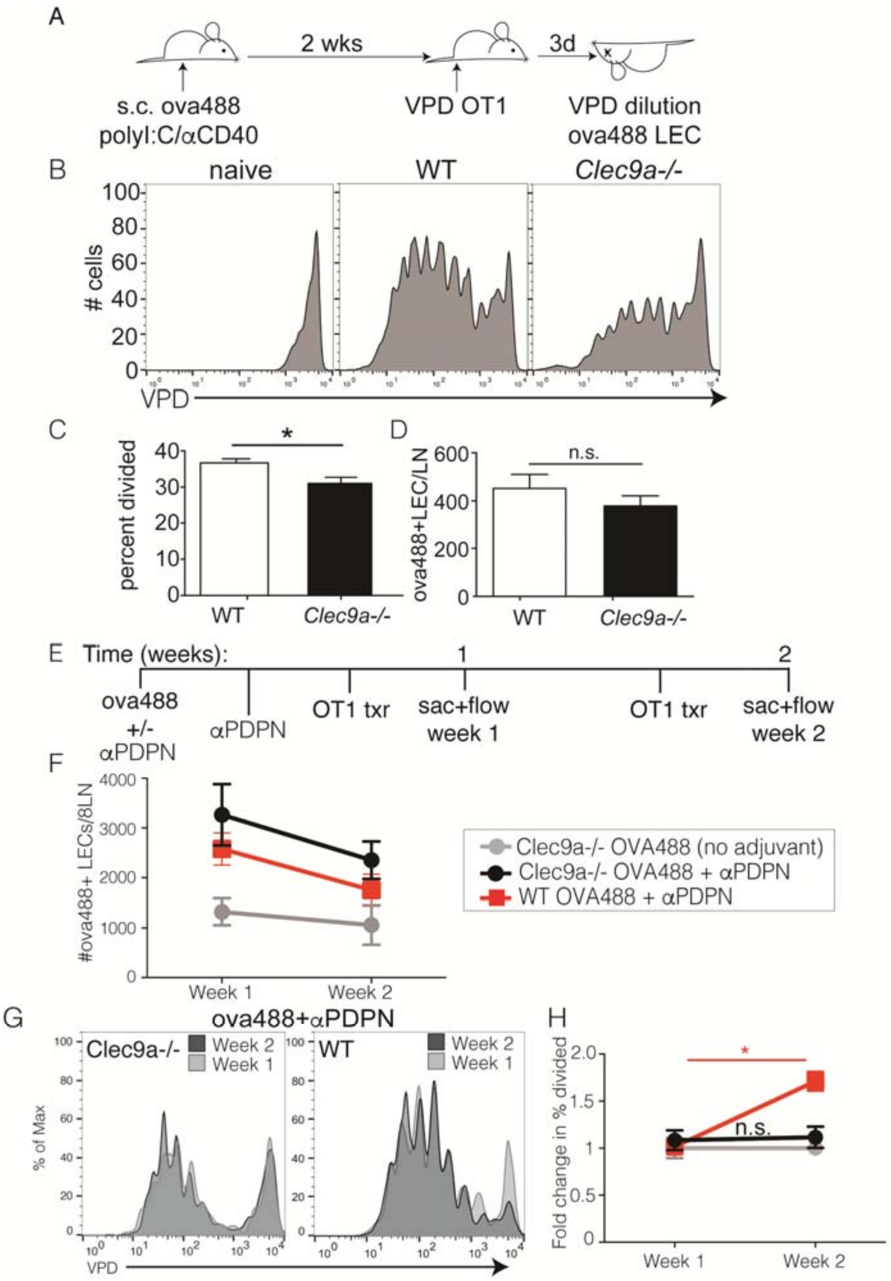

Supplementary figure 9. Clec9a is important for lymphatic endothelial cell – dendritic cell antigen exchange during lymph node contraction. A. Intact Wild-type or *Clec9a-/-* were immunized with ovalbumin conjugated to alexafluor 488 (ova488) (5μg/site), polyI:C (1μg/site) and αCD40 (1μg/site) and OT1 T cell transferred. Two weeks later mice were sacrificed and B. OT1 division was assessed and quantified in C. Shown are mice of each genotype pooled from 3 independent experiments of 3 mice each where the asterisk represents an unpaired t-test p-value of 0.01. D. Number of ova488+ lymphatic endothelial cells (LEC) was also calculated between groups with no significant difference by t-test. In all panels error bars shown in the figure are mean and SEM. E. Schematic for experimental design. F. Number of antigen positive LECs after expansion with 2 doses of anti-PDPN treatment in the first week and contraction of LECs at two weeks in either WT or *Clec9a-/-* mice and control *Clec9a-/-* without treatment. G. Examples of T cell division in PDPN treated WT or *Clec9a-/-* mice one or two weeks after anti-PDPN administration. H. Percent T cells divided was calculated to measure differences in the ability of *Clec9a-/-* mice to present antigen during LEC contraction. Statistical analysis was done using two-way ANOVA with a p-value of <0.05 being significant as shown by an asterisk or not significant (n.s.). Experiment was performed with 3 mice per group and repeated twice. Error bars are mean +/- standard error.
