# Supplementary material for: Migratory dendritic cells acquire and present lymphatic endothelial cell-archived antigens during lymph node contraction
Source: Nat Commun. 2017 Dec 11;8:2034. doi: 10.1038/s41467-017-02247-z (PMC5725486; doi:10.1038/s41467-017-02247-z)
Supplement: Supplementary file 3 — Description of Additional Supplementary Files [file 41467_2017_2247_MOESM3_ESM.pdf]

Description of Additional Supplementary Files

File Name: Supplementary Movie 1
Description: Non-antigen bearing lymphatic endothelial cells interact for shorter periods of time than antigen bearing lymphatic endothelial cells. Images of 39-42 xy planes (509µm x 509µm) with 3-µm z-spacing were acquired every minute for 30 min. Image analysis was performed using Imaris (Bitplane) and Matlab (Mathworks) software. The channel arithmetics function in Imaris was used to perform linear unmixing between colors using images from single color control lymph nodes acquired with the same instrument settings. Representative movie with a time lapse of 30 minutes where long term interactions between lymphatic endothelial cells (red) that are associated with antigen (green) and bone marrow derived dendritic cells (blue).

File Name: Supplementary Movie 2
Description: Antigen bearing lymphatic endothelial cells interact for longer periods of time than non-antigen bearing lymphatic endothelial cells. As in supplemental movie 1 except time-lapse shown is representative of short interactions between lymphatic endothelial cells (red) and bone marrow derived dendritic cells (blue) when antigen (green) is not present on the lymphatic endothelial cells.

File Name: Supplementary Movie 3
Description: Lymphatic endothelial cells and dendritic cells both interact with antigen. As in supplemental movie 1. Movie from images shown in figure 2 and supplementary figure 4.

File Name: Supplementary Movie 4
Description: Antigen specific T cells interact for with bone marrow derived dendritic cells in the T cell zone. As in supplemental movie 1 except T cells (white) are shown interacting with bone marrow derived dendritic cells (blue) in the center of the lymph node. Scale bars is 30μm.
